# Supplementary material for: Improvement of In-School Physical Activity with Active School-Based Interventions to Interrupt Prolonged Sitting: A Systematic Review and Meta-Analysis
Source: Int J Environ Res Public Health. 2023 Jan 16;20(2):1636. doi: 10.3390/ijerph20021636 (PMC9863961; doi:10.3390/ijerph20021636)
Supplement: Supplementary file 1 [file ijerph-20-01636-s001.zip › ijerph-2147676-supplementary.pdf]

PubMed

Search strategy: #1 AND #2 AND #3

#1 Population

("Child"[Mesh] OR "Adolescent"[Mesh])

#2 Behaviour

("Risk Reduction Behavior"[Mesh]) OR ("Sedentary Behavior"[Mesh]) OR ("Sitting Position"[Mesh]) OR ("Posture"[Mesh]) OR ("Posture/physiology"[Mesh]) OR ("Rest"[Mesh]) OR ("Rest/physiology"[Mesh]) OR ("Risk Factors"[Mesh]) OR ("Prolonged Sitting")

#3 Intervention

("Break*" OR "Break up" OR "breaking up" OR "Bout*" OR "Interrupt*")

Sport Discus

Search strategy: #1 AND #2 AND #3

#1 Population

(DE "CHILDREN") OR (DE "SCHOOL children") OR (DE "TEENAGERS") OR "child*" OR "adolescen*" OR "teen*" OR "youth*"

#2 Behaviour

(DE "SEDENTARY behavior in children") OR (DE "SEDENTARY behavior") OR (DE "HEALTH behavior") OR (DE "BEHAVIOR modification") OR (DE "SITTING position") OR (DE "SEDENTARY lifestyles") OR (DE "POSTURE") OR ("PROLONGED SITTING")

#3 Intervention

("BREAK**" OR "BREAK UP" OR "BREAKING UP" OR "BOUT*" OR "INTERRUPT*")

CINAHL

Search strategy: #1 AND #2

#1 Population and Behaviour

(MM "Life Style, Sedentary In Infancy and Childhood") OR (MM "Life Style, Sedentary In Adolescence") OR (MM "Sitting In Infancy and Childhood") OR (MM "Sitting In Adolescence") OR (MM "Posture In Infancy and Childhood") OR (MM "Posture In Adolescence") OR (MM "Health Behavior In Infancy and Childhood") OR (MM "Health Behavior In Adolescence") OR (MM "Behavior Modification In Infancy and Childhood") OR (MM "Behavior Modification In Adolescence") OR ("Prolonged Sitting")

#2 Intervention

(“Break*” OR “Break up” OR “breaking up” OR “Bout*” OR “Interrupt*”)

EMBASE

Search strategy: #1 AND #2 AND #3

#1 Population

'child'/exp OR 'child' OR 'children' OR 'adolescent'/exp OR 'adolescent' OR 'teenager'

#2 Behaviour

'risk reduction'/mj OR 'sitting'/mj OR 'activity, sedentary' OR 'posture, sitting' OR 'seated position' OR 'sedentary activity' OR 'sitting' OR 'sitting position' OR 'sitting posture' OR 'sedentary lifestyle'/mj OR 'sedentary time'/mj OR 'rest'/mj

#3 Intervention

break* OR 'break up' OR 'breaking up' OR 'bout*' OR 'interrupt*'

COCHRANE LIBRARY

Search strategy: #3 AND #10 AND #11

Population

- #1 MeSH descriptor: [Child] explode all trees
- #2 MeSH descriptor: [Adolescent] explode all trees
- #3 #1 OR #2

Behavior

- #4 MeSH descriptor: [Risk Reduction Behavior] explode all trees
- #5 MeSH descriptor: [Sedentary Behavior] explode all trees
- #6 MeSH descriptor: [Sitting Position] explode all trees
- #7 MeSH descriptor: [Rest] explode all trees
- #8 MeSH descriptor: [Risk Factors] explode all trees
- #9 Prolonged sitting
- #10 #4 OR #5 OR #6 OR #7 OR #8 OR #9

Intervention

- #11 (Break*) OR ("break-up") OR (breaking up time) OR (Bout*) OR (Interrupt*)

PEDro

Search strategy:

- Child* Sedentary behaviour Break*
- Adolescent* Sedentary behaviour Break*
- Child* Sitting Break*

- Adolescent* Sitting Break*
- Child* Posture Break*
- Child* Rest Break
- Child* Risk Factors Break*
- Child* Sedentary Behaviour Bout*
- Child* Sitting Bout*
- Adolescent* Sitting Bout*
- Child* Rest Bout*
- Adolescent* Rest Bout*
- Child* Prolonged sitting Bout*
- Child* Sedentary behaviour Interrupt*
- Adolescent* Sedentary behaviour Interrupt*
- Child* Sitting Interrupt*
- Child* Risk Factors Interrupt*

Web of Science

Search strategy: #1 AND #2 AND #3

#1 Population

TS=("child*" OR "pediat*" OR "paediat*" OR "kid" OR "kids" OR "prepubescen*" OR "prepuberty*" OR "teen*" OR "young*" OR "youth*" OR "minors*" OR "under ag*" OR "underag*" OR "juvenile*" OR "girl*" OR "boy*" OR "preadolesc*" OR "adolesc*")

#2 Behaviour

TS=(“Risk Behavior*” OR “lifestyle risk*” OR “sedent*” OR “sedentary behaviour*” OR “sedentary lifestyle*” OR “sedentary time*” OR “sitting” OR “sitting position*” OR “prolonged sitting” OR “sitting time*” OR “seated position*”)

#3 Intervention

TS=(“Break*” OR “Break up” OR “breaking up” OR “Bout*” OR “Interrupt*”)
